# Decreased bone turnover in HIV-infected children on antiretroviral therapy

**DOI:** 10.1007/s11657-018-0452-6

**Published:** 2018-04-05

**Authors:** Stephanie Shiau, Michael T. Yin, Renate Strehlau, Faeezah Patel, Ndileka Mbete, Louise Kuhn, Ashraf Coovadia, Stephen M. Arpadi

**Affiliations:** 1 Gertrude H. Sergievsky Center, College of Physicians and Surgeons, Columbia University, 630 W. 168th Street, PH 19-114, New York, NY 10032 USA; 2 Department of Epidemiology, Mailman School of Public Health, Columbia University, New York, NY USA; 3 Empilweni Services and Research Unit, Rahima Moosa Mother and Child Hospital, Department of Pediatrics and Child Health, Faculty of Health Sciences, University of the Witwatersrand, Johannesburg, South Africa; 4 Department of Medicine, College of Physicians and Surgeons, Columbia University, New York, NY USA; 5 Department of Pediatrics, College of Physicians and Surgeons, Columbia University, New York, NY USA

**Keywords:** Bone turnover markers, Pediatrics, HIV, Antiretroviral therapy, Immune activation

## Abstract

***Summary*:**

In this study, we evaluated the relationships between immune activation, bone turnover, and bone mass in virally suppressed HIV-infected children and HIV-uninfected children in South Africa. We found that decreased bone mass may occur or persist independent of immune activation and altered bone turnover.

**Purpose:**

HIV-infected children and adolescents have deficits in skeletal growth which include decreases in bone mass and alterations in bone microarchitecture. However, the mechanism by which HIV infection compromises bone accrual in children and adolescents is unclear. The goal of this study was to evaluate the relationships between immune activation, bone turnover, and bone mass in a group of pre-pubertal HIV-infected children randomized to remain on ritonavir-boosted lopinavir (LPV/r)-based antiretroviral therapy (ART) or switch to efavirenz-based ART in South Africa virally suppressed at the time of this study.

**Methods:**

This cross-sectional analysis included 219 HIV-infected and 180 HIV-uninfected children enrolled in the CHANGES Bone Study conducted in Johannesburg, South Africa. Whole body (WB) bone mineral content (BMC) was assessed by dual x-ray absorptiometry and WB BMC *Z*-scores adjusted for sex, age, and height were generated. Bone turnover markers, including C-telopeptide of type 1 collagen (CTx) and procollagen type I N-terminal propeptide (P1NP), were analyzed. Markers of immune activation were also measured, including cytokines IL-6 and TNF-alpha, as well as soluble CD14 and high-sensitivity C-reactive protein (CRP).

**Results:**

Compared to uninfected controls, HIV-infected children had lower WB BMC *Z*-scores, similar IL-6 and TNF-alpha, higher soluble CD14 and high-sensitivity CRP, and lower markers of bone resorption (CTX) and bone formation (P1NP). Bone turnover markers were not different in those remaining on LPV/r or switched to efavirenz.

**Conclusions:**

Our findings suggest that in HIV-infected children with viral suppression, decreased bone accrual may occur or persist independent of immune activation and altered bone turnover.

## Introduction

HIV-infected children and adolescents have deficits in skeletal growth which include decreases in bone mass accrual and alterations in bone microarchitecture [1, 2]. We previously reported lower bone mineral content (BMC) in a group of South African HIV-infected children who initiated antiretroviral therapy (ART) early in life before 2–3 years of age and have had excellent virologic control [3]. In particular, among children remaining on ritonavir-boosted lopinavir (LPV/r)-based ART, we observe lower accrued bone mass compared to children switching to efavirenz-based ART. This disruption of bone accrual during critical periods of skeletal development can compromise adult peak bone mass and increase the risk of later life osteoporosis and fracture [4, 5].

However, the mechanisms by which HIV infection compromises bone accrual in children and adolescents are not well defined. One hypothesized mechanism is that chronic immune activation associated with HIV infection, despite the use of ART, induces alterations in bone remodeling, i.e., increased bone resorption, decreased bone formation, or both, resulting in decreased bone acquisition during growth. In HIV-infected adults, higher serum levels of pro-resorptive cytokines and T cell activation markers are associated with lower bone mineral density (BMD) [6–9], suggestive of immune activation-mediated increased bone resorption. Due to the invasive nature of bone biopsy for performing histomorphometry, insights about mechanism are largely reliant on biochemical markers of bone turnover. In the few available studies of bone turnover markers in HIV-infected children, findings are inconsistent [10–12].

The primary goal of this study was to evaluate the relationships between immune activation, bone turnover, and bone mass in a group of well-suppressed pre-pubertal HIV-infected children in urban South Africa randomized to remain on LPV/r-based ART or switch to efavirenz-based ART [13, 14]. A better mechanistic understanding may assist development of effective interventions to optimize bone health during childhood.

## Methods

### Study population

Of 219 HIV-infected and 219 HIV-uninfected children enrolled in the CHANGES Bone Study conducted at Rahima Moosa Mother and Child Hospital in Johannesburg, South Africa [3], all 219 HIV-infected children and 180 (82.2%) of the HIV-uninfected children with available relevant measurements are included in this cross-sectional analysis of bone turnover and immune activation. The 219 HIV-infected children were previous participants in a non-inferiority randomized clinical trial evaluating the safety and efficacy of preemptive switching to efavirenz compared with remaining on LPV/r [13, 14].

### Measurements and procedures

At the study visit, demographic data were collected and all participants underwent physical examinations to obtain anthropometric measures and to assess pubertal development. For HIV-infected children, plasma HIV-RNA levels (lower limit of detection 40 copies/mL) were measured by the Abbott RealTime HIV-1 Assay (Abbott Park, Illinois, USA). CD4 counts and percentage were measured by the TruCount Method (BD Biosciences, Germany).

Additional plasma was stored at − 80 °C and shipped to the Biomarkers Core Laboratory, Irving Institute for Clinical and Translational Research, at Columbia University Medical Center in New York, NY, USA, where they were analyzed for bone turnover markers, including C-telopeptide of type 1 collagen (CTx) (ELISA; Immunodiagnostic Systems, Scottsdale, AZ, USA) and procollagen type I N-terminal propeptide (P1NP) (RIA; Immunodiagnostic Systems, Scottsdale, AZ). Markers of immune activation were also analyzed, including pro-inflammatory, pro-resorptive cytokines interleukin-6 (IL-6) (ELISA; R&D Systems, Minneapolis, MN) and TNF-alpha (ELISA; R&D Systems, Minneapolis, MN, USA), as well as soluble CD14 (ELISA; R&D Systems, Minneapolis, MN, USA), a marker of monocyte activation, and high-sensitivity C-reactive protein (CRP) (Cobas Integra 400 Plus; Roche Diagnostics, Indianapolis, IN, USA), an acute phase reactant and marker of general inflammation. In addition, plasma was analyzed for intact parathyroid hormone (iPTH) (RIA; Scantibodies Laboratory, Santee, CA, USA), serum 25-hydroxyvitamin D3 (25(OH)D_3_) (LCMS; Agilent, Santa Clara, CA, USA), creatinine (Cobas Integra 400 Plus; Roche Diagnostics, Indianapolis, IN), and cystatin C (Cobas Integra 400 Plus; Roche Diagnostics, Indianapolis, IN, USA). Given limited reference values on markers of immune activation, renal function, and bone turnover for children, reference ranges provided by the Biomarkers Core Laboratory were utilized.

Whole body (WB) and lumbar spine (LS) dual x-ray absorptiometry (DXA) scans were obtained using a single Hologic scanner and data was transferred to the Image Reading Center in New York for reading. WB and LS BMC *Z*-scores adjusted for sex, age, and height based on reference normative values from the United States Bone Mineral Density in Childhood Study were generated [15], as there are no South African or African reference ranges available. To account for potential effects of growth velocity on bone turnover markers, we assessed growth velocity (cm/year) for HIV-infected children by calculating the change in height over time between the date of DXA scan and the previous study visit [16].

Signed informed consent was provided by each child’s parent or guardian; children provided assent if they were at least 7 years old and deemed able to understand. The study was approved by the Institutional Review Boards of Columbia University (New York, NY, USA) and the University of the Witwatersrand (Johannesburg, South Africa).

### Statistical analysis

Chi-squared or Fisher’s exact tests were used to compare proportions, *t* tests to compare means, and Wilcoxon rank-sum test to compare medians. Linear regression was first used to evaluate the association between HIV and bone turnover markers, adjusted for age, sex, 25(OH)D_3_, and WB bone area. Given potential differences in the number of osteoblasts between groups, we included WB bone area as a proxy to account for this in the association between HIV and bone turnover markers, assuming the groups had no difference in the number of osteoblasts per surface area [17]. Next, linear regression was used to evaluate the association between bone turnover markers and WB BMC and LS BMC *Z*-scores and between markers of immune activation and bone turnover markers, adjusted for age and sex. Linear regression analyses were also conducted among HIV-infected children evaluating the associations between treatment regimen and bone turnover markers, adjusted for age, sex, and 25(OH)D_3_ and, as bone turnover markers vary with growth velocity as well as bone surface area [17], growth velocity and WB bone area were also included. All *p* values are two-tailed and *p* values < 0.05 were considered statistically significant. All statistical calculations were performed using SAS version 9.4 (Cary, North Carolina, USA).

## Results

### Characteristics

Characteristics of the study participants are shown in Table 1. The 219 HIV-infected children (49% male) and 180 HIV-uninfected children (55% male) were between 5 and 9 years of age (mean 6.7 years). One hundred thirteen HIV-infected children were previously randomized to remain on LPV/r and 106 were randomized to switch to efavirenz. Children were also receiving two nucleoside reverse transcriptase inhibitors including lamivudine and either abacavir, zidovudine, or stavudine. None had any past or current use of tenofovir and none was receiving corticosteroids or antiepileptic medications. The mean (SD) duration on treatment for HIV-infected children was 5.7 (1.1) years (range 2.8–8.7) and this visit occurred 1–4 years (mean 2.1 years) after randomization in the clinical trial. At the time of evaluation, 93.6% of children had HIV-1 RNA < 400 copies/mL and a mean (SD) CD4 percentage of 37.3 (7.1).

**Table 1:** Characteristics of 219 HIV-infected and 180 HIV-uninfected children in Johannesburg, South Africa

Characteristic	HIV+ (*N* = 219)	HIV− (*N* = 180)	*P*	LPV/r (*N* = 113)	EFV (*N* = 106)	*P*
Male, *N* (%)	107 (48.9)	99 (55.0)	0.22	53 (46.9)	54 (50.9)	0.55
Age, mean (SD)	6.4 (1.2)	7.1 (1.6)	< 0.001	6.4 (1.3)	6.3 (1.2)	0.74
Tanner stage 1, *N* (%)	215 (98.2)	172 (95.6)	0.128	113 (100.0)	102 (96.2)	0.053
Weight in kg, mean (SD)	19.2 (3.9)	22.7 (5.6)	< 0.0001	19.0 (3.6)	19.4 (4.1)	0.49
WAZ, mean (SD)	− 0.83 (0.9)	− 0.32 (1.1)	< 0.0001	− 0.90 (0.9)	− 0.76 (0.9)	0.25
Underweight, *N* (%)	24 (11.0)	6 (3.3)	0.004	15 (13.3)	9 (8.5)	0.26
Height in cm, mean (SD)	110.3 (8.3)	117.2 (9.9)	< 0.0001	111 (8.6)	110 (8.0)	0.51
HAZ, mean (SD)	− 1.40 (0.9)	− 0.87 (0.92)	< 0.0001	− 1.36 (0.9)	− 1.45 (0.9)	0.48
Stunted (HAZ < − 2), *N* (%)	61 (27.9)	18 (10.0)	< 0.0001	30 (26.6)	31 (29.3)	0.66
Body mass index (BMI), mean (SD)	15.7 (1.6)	16.3 (2.2)	0.0008	15.4 (1.4)	15.9 (1.7)	0.018
BAZ, mean (SD)	0.08 (1.0)	0.28 (1.1)	0.049	− 0.05 (0.9)	0.21 (1.0)	0.046
Age at ART start in months, mean (SD)	8.8 (6.8)			9.2 (6.7)	8.5 (6.8)	0.49
Plasma HIV-1 RNA < 400 copies/mL, *N* (%)	205 (93.6)			105 (92.9)	100 (94.3)	0.67
CD4 percentage, mean (SD)	37.3 (7.1)			35.7 (6.6)	39.0 (7.2)	0.0006
Treatment duration in years, mean (SD)	5.7 (1.1)			5.7 (1.1)	5.7 (1.1)	0.98
Time since randomization in years, mean (SD)	2.1 (0.6)			2.2 (0.6)	2.1 (0.6)	0.54
Remained on randomized regimen at time of bone assessment, *N* (%)	201 (91.8)			102 (90.3)	99 (93.4)	0.40
Whole body BMC *Z*-score	− 0.95 (0.83)	− 0.79 (0.78)	0.05	− 1.20 (0.82)	− 0.68 (0.76)	< 0.001
Lumbar spine BMC *Z*-score	− 0.22 (0.89)	− 0.38 (0.83)	0.08	− 0.45 (0.89)	0.01 (0.84)	0.0001
25(OH)D_3_ (ng/mL), mean (SD) Missing	30.6 (9.8) 6	24.3 (6.3) 2	< 0.0001	34.0 (10.0) 2	26.9 (8.1) 4	< 0.0001
25(OH)D_3_ < 20 ng/mL, *N* (%)	27 (12.7)	44 (24.7)	0.002	8 (7.2)	19 (18.6)	0.012
iPTH (pg/mL) Missing	31.1 (12.9) 3	32.1 (15.7) 3	0.50	30.7 (12.1) 1	31.5 (13.7) 2	0.62
iPTH ≥ 65 pg/mL, *N* (%)	3 (1.4)	7 (4.0)	0.11	2 (1.8)	1 (1.0)	0.61
Cystatin C (mg/L) Missing	0.68 (0.10) 4	0.72 (0.10) 2	< 0.0001	0.70 (0.11) 1	0.67 (0.09) 3	0.029
Cystatin C ≥ 0.95 mg/L, *N* (%)	5 (2.3)	2 (1.1)	0.37	4 (3.6)	1 (1.0)	0.21
Creatinine (mg/dL) Missing	0.38 (0.07) 4	0.44 (0.08) 3	< 0.0001	0.38 (0.08) 1	0.38 (0.07) 3	0.96
Creatinine ≥ 1.20 mg/dL, *N* (%)	0 (0.0)	0 (0.0)	1.0	0 (0.0)	0 (0.0)	1.0

*Z*-scores from Bone Mineral Density in Childhood Study (adjusted for age, sex, race, and height-for-age *Z*-score)

*BMC* bone mineral content, *BMD* bone mineral density, *WAZ* weight-for-age *Z*-score, *HAZ* height-for-age *Z*-score, *BAZ* BMI-for-age *Z*-score, *iPTH* intact parathyroid hormone, *LPV/r* ritonavir-boosted lopinavir, *EFV* efavirenz, *iPTH* intact parathyroid hormone, *25(OH)D*_3_ serum 25-hydroxyvitamin D3

HIV-infected children had lower mean WB BMC *Z*-score compared with the HIV-uninfected group (− 0.95 vs. − 0.79, *p* = 0.05), similar to the result previously reported [3]. LS BMC *Z*-score was not different between groups (− 0.22 vs. − 0.38, *p* = 0.08). The mean concentration of 25(OH)D_3_ was higher in HIV-infected children than in HIV-uninfected children (30.6 vs. 24.3 ng/mL, *p* < 0.01). A higher proportion of HIV-infected children had 25(OH)D_3_ > 20 ng/mL (84.9 vs. 74.4%, *p* = 0.009). Mean iPTH concentration was similar in the groups (31.1 vs. 32.1 pg/mL, *p* = 0.5). The mean cystatin C and creatinine levels were lower in the HIV-infected group, but overall, few HIV-infected or HIV-uninfected children had elevated cystatin C (Table 1). HIV-infected children randomized to remain on LPV/r had lower WB BMC and LS BMC *Z*-score compared to HIV-infected children randomized to switch to efavirenz. Those switched to efavirenz had a significantly lower mean 25(OH)D_3_ concentration than those remaining on LPV/r (26.9 vs. 34.0 ng/mL, *p* < 0.001), as well as a greater proportion with 25(OH)D_3_ < 20 ng/mL (18.6 vs. 7.2%, *p* = 0.012).

### Immune activation

As shown in Table 2, mean IL-6 concentration was similar in HIV-infected and HIV-uninfected children; only seven children (four HIV-infected and three HIV-uninfected) had elevated IL-6. Although mean TNF-alpha was slightly lower in the HIV-infected group, results for most children fell well within the reference range provided by the Biomarkers Core Laboratory (< 4.71 pg/mL). Mean soluble CD14 concentration was higher in the HIV-infected group compared to the HIV-uninfected group (1453 vs. 1195 ng/mL, *p* < 0.0001) and 8.3% of the HIV-infected children had an elevated soluble CD14 ≥ 2300 ng/mL compared to 2.2% of the HIV-uninfected children (*p* = 0.005). Similarly, mean high-sensitivity CRP was higher in the HIV-infected group compared to the HIV-uninfected group (4.75 vs. 1.81 mg/dL, *p* = 0.008) and a higher proportion of the HIV-infected children had an elevated high-sensitivity CRP concentration ≥ 0.5 mg/dL than the HIV-uninfected children (59.4 vs. 43.3%, *p* = 0.002).

**Table 2:** Markers of immune activation of 219 HIV-infected and 180 HIV-uninfected children in Johannesburg, South Africa

Measurement	HIV+ (*N* = 219)	HIV− (*N* = 180)	*P*	LPV/r (*N* = 113)	EFV (*N* = 106)	*P*
IL-6 (pg/mL), mean (SD) Missing	1.72 (3.6) 0	1.73 (3.49) 0	0.97	2.17 (4.8) 0	1.25 (1.4) 0	0.059
IL-6 ≥ 9.96 pg/mL, *N* (%)	4 (1.8)	3 (1.7)	0.66	4 (3.5)	0 (0.0)	0.051
TNF-alpha (pg/mL), mean (SD) Missing	2.15 (1.35) 0	2.60 (1.21) 1	0.0008	2.40 (1.33) 0	1.89 (1.34) 0	0.005
TNF-alpha ≥ 4.71 pg/mL, *N* (%)	6 (2.7)	9 (5.0)	0.23	4 (3.5)	2 (1.9)	0.45
Soluble CD14 (ng/mL), mean (SD) Missing	1453 (550) 1	1195 (437) 0	< 0.0001	1428 (535) 0	1480 (566) 1	0.493
Soluble CD14 ≥ 2300 ng/mL, *N* (%)	18 (8.3)	4 (2.2)	0.005	10 (8.9)	8 (7.6)	0.61
High-sensitivity C-reactive protein (mg/dL), mean (SD) Missing	4.00 (10.4) 5	1.81 (4.02) 2	0.008	4.75 (12.6) 2	3.2 (7.5) 3	0.28
High-sensitivity C-reactive protein ≥ 0.5 mg/dL, *N* (%)	127 (59.4)	77 (43.3)	0.002	64 (56.6)	64 (60.4)	0.57

*IL-6* interleukin-6, *TNF-alpha* tumor necrosis factor alpha, *LPV/r* ritonavir-boosted lopinavir, *EFV* efavirenz

The HIV-infected children randomized to remain on LPV/r had a non-significantly higher mean IL-6 concentration compared to those switched to efavirenz (2.17 vs. 1.25 pg/mL, *p* = 0.059); four (1.8%) in the LPV/r group and zero (0%) in those switched to efavirenz had concentrations exceeding the upper limit of the reference range. In addition, those on LPV/r had a higher mean TNF-alpha concentration compared to those switched to efavirenz (2.40 vs. 1.89 pg/mL, *p* = 0.005). Four in the LPV/r group and two in the group switched to efavirenz had TNF-alpha concentrations that exceeded the upper limit of the reference range. The mean soluble CD14 and high-sensitivity CRP concentrations were similar in the two treatment groups.

### Bone turnover markers

As shown in Table 3, CTX (1.72 vs. 2.05 ng/mL, *p* < 0.01) and P1NP (584 vs. 634 ng/mL, *p* < 0.01) concentrations were lower in HIV-infected than in HIV-uninfected children. CTX and P1NP remained lower for the HIV-infected group after adjusting for age, sex, 25(OH)D_3_, and WB bone area. CTX and P1NP concentrations for most HIV-infected and HIV-uninfected children fell within the reference ranges provided by the Biomarkers Core Laboratory: 93.6% of HIV-infected children and 94.4% of the HIV-uninfected children had a CTX level between 0.83 and 3.32 ng/mL; 88.6% of the HIV-infected group and 86.1% of the HIV-uninfected group had a P1NP level between 19 and 830 ng/mL. Among HIV-infected children, CTX and P1NP levels did not differ between children randomized to remain on LPV/r and children randomized to switch to efavirenz at a mean of 2.1 years after randomization (Table 3), nor were differences apparent after adjusting for age, sex, 25(OH)D_3_, growth velocity, and WB bone area. There were no associations between bone turnover markers and WB BMC and LS BMC *Z*-scores.

**Table 3:** Bone turnover markers of 219 HIV-infected and 180 HIV-uninfected children in Johannesburg, South Africa

Measurement	HIV+ (*N* = 219)	HIV− (*N* = 180)	*P*	LPV/r (*N* = 113)	EFV (*N* = 106)	*P*
CTX (ng/mL), mean (SD)	1.72 (0.63)	2.05 (0.69)	< 0.0001	1.70 (0.63)	1.75 (0.64)	0.53
CTX (ng/mL), median (IQR)	1.67 (1.22, 2.10)	2.05 (1.55, 2.55)	< 0.0001	1.67 (1.22, 2.04)	1.67 (1.24, 2.19)	0.57
CTX (ng/mL), *N* (%)						
< 0.83	12 (5.5)	5 (2.8)	0.16	6 (5.3)	6 (5.7)	0.99
0.83–3.32 (normal)	205 (93.6)	170 (94.4)		106 (93.8)	99 (93.4)	
≥ 3.32	2 (0.9)	5 (2.8)		1 (0.9)	1 (0.9)	
P1NP (ng/mL), mean (SD)	584 (183)	634 (173)	0.005	585 (179)	583 (188)	0.94
P1NP (ng/mL), median (IQR)	564 (454, 680)	616 (522, 719)	0.002	560 (469, 686)	566 (454, 664)	0.76
P1NP (ng/mL), *N* (%)						
< 190	1 (0.5)	0 (0.0)	0.53	1 (0.9)	0 (0.0)	1.0
190–830 (normal)	194 (88.6)	155 (86.1)		100 (88.5)	94 (88.7)	
≥ 830	24 (11.0)	25 (13.9)		12 (10.6)	12 (11.3)	

*LPV/r* ritonavir-boosted lopinavir, *EFV* efavirenz

### Relationships between markers of immune activation and bone turnover markers in HIV-infected children

Next, we evaluated relationships between markers of immune activation and bone turnover markers among our HIV-infected children. None of the markers of immune activation were associated with CTX. Although soluble CD14 and TNF-alpha were not associated with P1NP, IL-6 was negatively associated with P1NP (*β* = − 0.87, SE = 0.34, *p* = 0.01), even after adjustment for age and sex. Similarly, high-sensitivity CRP was negatively associated with P1NP (*β* = − 3.6, SE = 1.2, *p* = 0.002), even after adjustment for age and sex. Stratifying these relationships by treatment group (LPV/r or efavirenz) resulted in similar findings (data not shown).

## Discussion

In this study of South African HIV-infected children on ART with mainly undetectable viral load on long-term effective ART, we found lower bone mass, limited evidence of immune activation, and lower measures of bone remodeling compared to HIV-uninfected children.

Though our bone turnover markers were lower in HIV-infected than in HIV-uninfected children, both groups fell within the range reported in other studies of bone turnover in children without HIV [16, 18]. Our finding of less bone remodeling among HIV-infected children differs from results reported in older studies of HIV-infected children and adolescents [11, 12, 19, 20]. These differences may be explained by varied study population characteristics with respect to level of viral suppression, duration and type of ART exposure, and differences in specific bone turnover markers assayed. We were unable to have the children fast for the blood measurement due to travel times to the study site and the age of the children. Mora et al. found that HIV-infected children and adolescents aged 6–17 years on ART, including indinavir, nelfinavir, and ritonavir-based regimens, had higher bone formation (bone alkaline phosphatase, BALP) as well as higher bone resorption (N-telopeptide, NTX) than HIV-uninfected children [11]. Similarly, Tan et al. reported higher bone formation, as measured by osteocalcin, in HIV-infected children on protease inhibitors ages 2 to 12 years, than HIV-uninfected controls [12]. In contrast to these two studies in which children were receiving a variety of older protease inhibitors (e.g., indinavir and nelfinavir) and nucleoside reverse transcriptase inhibitors (e.g., didanosine and stavudine), children in our study were initiated and suppressed early in life with currently recommended three-drug regimens. Over 90% of children in our study were virally suppressed, while only 40% of the participants in Tan et al. had HIV RNA < 400 copies/mL at the time of study.

More recently, a European study of 54 HIV-infected children and adolescents aged 6–19 years reported that HIV-infected children had higher bone formation (BALP) than controls, possibly due to lower serum concentrations of sclerostin and Dkk1, inhibitors of the Wnt/b-catenin pathway, a critical regulator of skeletal development [19]. Another study of adolescents aged 10–18 years in Thailand found increased bone turnover markers to be inversely associated with BMD *Z-*scores [20]. Differences in findings among publications may also be attributable to participant age and maturity, as well as variable time to ART initiation after HIV infection. For example, there was no age overlap between our study (5–9 years) and Sudjaritruk et al., and all but four HIV-infected children in our study were Tanner stage 1 whereas 73% of the population in Sudjaritruk et al. was Tanner stages 3–5. Of note, we observed low bone formation in a setting of no tenofovir use.

Also unexpected, we did not observe increased levels of pro-inflammatory pro-resorptive cytokines (IL-6 and TNF-alpha) in our HIV-infected group. This may be due to ART initiation early in life and well-controlled virus for an extended period of time. In addition, the HIV-infected children had higher vitamin D than the HIV-uninfected children which is likely due to greater proportion of children with HIV receiving multivitamin supplements; vitamin D has recently been shown to attenuate immune activation in youth with HIV [21]. Aside from a weak negative association between IL-6 and P1NP, no evidence of immune activation-mediated increased bone resorption, as seen in other studies of children and adults, was seen [9, 22]. Also observed were increased levels of soluble CD14, a marker of monocyte activation, in our HIV-infected group. Similarly, higher levels of plasma soluble CD14 at longitudinal time points following viral load suppression and normalization of CD8+ T cell activation have been reported in a study of HIV-infected children in Vietnam [23]. In contrast to findings from a cross-sectional study of adolescents ages 14–25 years by Ruan et al., we did not observe a significant inverse correlation between soluble CD14 and our measures of bone mass [24]. Ruan et al. measured soluble CD14 and bone mass early in infection (2–3 years); although HIV-associated monocyte activation does not appear to be responsible for the decreased bone accrual we observe among HIV-infected children in our study, it may play a role closer to the time of infection or during periods of viremia. Persistent monocyte activation may be of great concern for HIV-infected children, since soluble CD14 has been proposed as an independent predictor of mortality as well as subsequent non-AIDS events (e.g., cardiovascular disease) in adults with HIV [25, 26]. Of note, high-sensitivity CRP levels were elevated in 59.4% of the HIV-infected children and 43.3% of the HIV-uninfected children, possibly reflecting elevated levels of general inflammation in this setting.

Although we saw differences in bone mass between children remaining on LPV/r and children switched to efavirenz, bone turnover markers did not discriminate between the groups, and both treatment groups (i.e., LPV/r and efavirenz) had lower bone turnover markers compared to HIV-uninfected children. The null finding did not appear to be explained by growth velocity. As expected, we observed lower vitamin D levels in the children switched to efavirenz. Efavirenz has been implicated in induction of 24-hydroxylase (CYP24A1), a cytochrome P450 enzyme that degrades 25(OH)D and 1,25(OH)_2_D into an inactive form [27]. The significance of this to bone metabolism is unclear given that iPTH concentrations were not elevated among those switched to efavirenz nor did they have lower BMC. Among the 19 HIV-infected children in the efavirenz group with low vitamin D (25(OH)D_3_ < 20 ng/mL), iPTH ranged from 13 to 107 pg/mL, with a mean of 36.0 pg/mL, and few above 65 pg/mL.

Children remaining on LPV/r had higher levels of pro-inflammatory, pro-resorptive cytokines IL-6 and TNF-alpha compared to children switched to efavirenz. This may be a direct effect of lopinavir and ritonavir, which in ex vivo studies in macrophages induce IL-6 and TNF-alpha expression via ERK signaling pathways and unfolded protein response [28]. Nonetheless, the relationship between markers of immune activation and bone turnover markers was not different between children on LPV/r and children on efavirenz. Furthermore, no relationship between the bone turnover markers and bone were identified. It is possible that the differences in bone mass between the treatment groups could have been related to differences in bone turnover that occurred close to the time of randomization that are no longer detectable. Future studies closer to the time of treatment switch may help clarify this.

ART initiation studies in adults suggest that the greatest declines in BMD occur around the time of ART initiation, likely due to a combination of direct ART effect and immune activation-mediated bone resorption [29, 30]. Our findings suggest that in HIV-infected children with viral suppression on stable ART, decreases in BMD persist independent of immune activation and altered bone turnover. This study takes place well beyond the time of initial viral suppression and is limited by a single measurement of bone turnover markers and markers of immune activation, which vary throughout childhood lack established reference ranges [18, 31]. Longitudinal studies that include serial measures of immune activation, bone turnover, and BMD are necessary to fully appreciate the dynamics of immune activation and bone modeling and remodeling in growing children with HIV.
